# HMGB-1 Increases Proinflammatory Reaction via TLR4 in Human Granulosa Cells of Endometriosis

**DOI:** 10.3390/jcm14217532

**Published:** 2025-10-24

**Authors:** Hye In Kim, Kyung Hee Kim, SiHyun Cho, Young Sik Choi, Byung Seok Lee, Seung Joo Chon, Bo Hyon Yun

**Affiliations:** 1 Department of Obstetrics and Gynecology, Yongin Severance Hospital, Yonsei University College of Medicine, Yongin 16995, Republic of Korea; hyein24@yuhs.ac; 2 Institute of Women’s Life Medical Science, Yonsei University College of Medicine, Seoul 03722, Republic of Korea; 3 Department of Obstetrics and Gynecology, Gangnam Severance Hospital, Yonsei University College of Medicine, Seoul 06273, Republic of Korea; 4 Department of Obstetrics and Gynecology, Gil Hospital, Graduate School of Medicine, Gachon University of Medicine and Science, Inchon 21565, Republic of Korea

**Keywords:** HMGB-1, granulosa cell, endometriosis, oxidative stress

## Abstract

**Background/Objectives:** Oxidative stress is a critical factor in the development and progression of endometriosis. Granulosa cells, which reside near oocytes in follicles, exhibit steroidogenic activity, and, consequently, influence oocyte quality. Increased oxidative stress may induce the danger signal such as HMGB-1 in granulosa cells and eventually change the follicular environment of patients with endometriosis. This study aimed to demonstrate that HMGB-1 and its receptors, TLR4 and RAGE, play important roles in the changes in the follicular environment in infertile patients with endometriosis. **Methods**: In the immortalized human granulosa cell line (hGL5), cell proliferation and apoptosis assay, ELISA for estradiol, qRT-PCR for HMGB-1 and TLR4, Western blot for apoptosis-related and NF-κB pathway-related proteins, and ELISA for inflammatory molecules IL-1β and IL-6 were performed after H_2_O_2_ treatment. **Results**: H_2_O_2_ treatment to the hGL5 cell line decreased cell proliferation via apoptosis and, as a result, decreased steroidogenesis. Also, it increased the gene expression of HMGB-1 and TLR4, increased the protein expression related to the NF-κB pathway, and increased the release of inflammatory molecules IL-1β and IL-6. **Conclusions**: The results indicate that oxidative stress associated with endometriosis may increase inflammation by interacting with HMGB-1 and TLR4 and activating the NF-κB pathway to increase proinflammatory responses. The findings of this study may provide insight into endometriosis with decreased oocyte quality.

## 1. Introduction

Endometriosis (EMS) is an inflammatory disease characterized by endometrial tissue outside the endometrium and myometrium. As with other gynecologic diseases, diagnosis of EMS is made by integrating symptoms with various imaging studies including ultrasound, CT, and MRI, along with blood test results [1]. Up to 50% of the women with pelvic pain or infertility are affected by EMS [2,3]. Both primary and secondary infertility are highly prevalent in EMS patients [4]. Elevated levels of inflammatory cytokines in the pelvic cavity of EMS patients suggest that inflammation and immune dysregulation are related to the establishment of EMS [5].

Oxidative stress results from an imbalance between reactive oxygen species and antioxidants. It has been widely accepted that oxidative stress contributes to the pathophysiology and progression of EMS by promoting local inflammation within the pelvic cavity [6]. Activated macrophages and endometrial tissue transplanted into the pelvic cavity induce oxidative stress [7]. The increased oxidative stress also alters the follicular microenvironment and is suspected to negatively affect the entire process of oocyte development [8].

Granulosa cells, which form multiple layers surrounding oocytes in follicles, influence the oocyte through direct gap junctions and play crucial roles in steroid hormone synthesis. As a result, they determine the oocyte competence and the infertility [9]. To understand the function and underlying mechanism of granulosa cells, selecting the appropriate ovarian granulosa cell line is important. The in vitro system lacks confounding factors such as paracrine factors and, therefore, is optimal to observe receptor functions at the molecular level.

The immortalized human granulosa cell line (hGL5), a well-established human granulosa cell line, demonstrates a consistent steroidogenic pathway and exhibits high proliferation rates, making it an optimal model for investigating cell survival, death, and steroidogenic activity [10]. The study with the hGL5 cell line can contribute to investigating infertility [11]. To explore infertility in EMS patients, it is crucial to understand the impact of oxidative stress on granulosa cells due to EMS. Kolesarova et al. used hGL5 cells as an in vitro model to study oxidative stress in ovarian granulosa cells [12]. Isoquercitrin, a dietary flavonoid, was shown to reduce H_2_O_2_-induced reactive oxygen species in hGL5 cells, demonstrating the model’s relevance for oxidative damage research. Additionally, Yun et al. reported that H_2_O_2_ treatment increases HMGB-1 and TLR4 expression in endometrial cells [13].

High-mobility group box-1 (HMGB-1) is a member of the chromosomal protein superfamily and acts as an inflammatory cytokine that can bind to DNA [14]. When passively released into the extracellular space, HMGB-1 functions as a damage-associated molecular pattern (DAMP) [15]. It has been reported that HMGB-1 expression is increased by oxidative stress-induced cell death in human endometrial stromal cells (HESCs) from patients with EMS [13]. HMGB-1 interacts with toll-like receptor 4 (TLR4) and activates inflammatory pathways such as the NF-κB pathway, thereby inducing alterations in eutopic endometrium and contributing to the development of EMS [16].

In the present study, we aimed to determine whether oxidative stress decreases the granulosa cell proliferation by inducing apoptosis and increases the release of HMGB-1, which in turn promotes inflammation via TLR4 and NF-κB pathways, ultimately lowering the quality of follicle in patients with EMS.

## 2. Materials and Methods

All experiments were performed using hGL5 cell line. The research protocol was approved by the Institutional Review Board of the Gachon University of Medicine and Science (GAIRB 2017-250).

### 2.1. hGL5 Cell Culture

The immortalized human granulosa cell line hGL5 was kindly provided by Professor Casarini (University of Modena and Reggio Emilia, Moderna, Italy). It was cultured in medium supplemented with 10% fetal bovine serum (FBS), 2% ultrasonic G, 2 mM L-glutamine, 100 U/mL penicillin, and 100 mg/mL streptomycin and maintained in an incubator at 37 °C and 5% CO_2_.

### 2.2. CCK-8 Assay

hGL5 cells were seeded at density of 1 × 10^5^ cells/well in six-well tissue culture plate. After 24 h, the culture medium was replaced with Dulbecco’s Modified Eagle Medium/Nutrient Mixture F-12 (DMEM/F12) containing 2% FBS. The cells were treated with 100 or 200 μM of H_2_O_2_ for 2 h to induce oxidative stress. Then 100 μL of CCK-8 reagent from Cell Counting Kit-8 (CCK-8, Dojindo, Kumamoto, Japan) was added to each well, and the cells were incubated at 37 °C for 24 h to evaluate cell proliferation. The supernatants were transferred to 96-well plate, and optical density at 450 nm was determined using VersaMax reader (Molecular Devices, Sunnyvale, CA, USA). The observance at 450 nm optical density was obtained.

### 2.3. Apoptosis Assay

After treatment with 100 or 200 μM of H_2_O_2_ for 2 h, 1 × 10^5^ cells/well of hGL5 cells in 6-well plates were incubated in serum-free medium for 24 h. The cells were harvested and washed once with cold phosphate-buffered saline (PBS) and once with binding buffer (10 mM Hydroxyethyl piperazine Ethane Sulfonicacid, pH 7.4, 140 mM NaCl, 2.5 mM CaCl_2_) from Fluorescein isothiocyanate (FITC) Annexin V Apoptosis Detection Kit (BD Pharmingen, BD Biosciences, San Jose, CA, USA). They were resuspended (1 × 10^6^ cells/mL) in the binding buffer. Then, 100 µL of the cell suspension (1 × 10^5^ cells) was transferred to 5 mL culture tube and incubated with five μL FITC-conjugated annexin V (AV) and 10 μL propidium iodide (PI) for 15 min at 25 °C in the dark. Next, the apoptosis was measured within 1 h using FACScan analyzer (Becton Dickinson and Company, Franklin Lakes, NJ, USA) according to the manufacturer’s instructions.

The apoptotic cells were defined as those stained positive for AV. The percentage of apoptotic cells, or the sum of cells in the early and late stages of apoptosis out of the entire cells, was calculated.

### 2.4. Western Blot

For Western blots for apoptosis-related proteins and HMGB-1, hGL5 cells were treated with different concentrations of H_2_O_2_ for 2 h and incubated for 24 h in serum-free DMEM. For Western blot for NF-κB pathway-related proteins and TLR4, hGL5 cells were treated with 200 μM of H_2_O_2_ for 2 h and incubated for 0 min, 15 min, 30 min, and 60 min in serum-free DMEM.

For both experiments, hGL5 cells were harvested and lysed using radioimmunoprecipitation analysis buffer (RIPA buffer; iNtRON Biotechnology, Seongnam, Republic of Korea) with protease inhibitor cocktail (Cell Signaling Technology, Beverly, MA, USA). After mixing, the cell lysates were distributed in 40 μL aliquots. The lysates were then centrifuged for 30 min at 13,000 rpm at 4 °C. Proteins were collected from the supernatant, and the protein concentration was determined using bicinchoninic acid protein analysis kit (Thermo Scientific, Hudson, NH, USA).

Western blot was performed as follows: 30 μg of each cell lysate were added to 5× buffer and boiled. After centrifugation, the supernatant was filtered, and sodium dodecyl sulfate-polyacrylamide gel electrophoresis (SDS-PAGE) was performed. The samples were loaded onto 8% SDS-PAGE gels and transferred to polyvinylidene fluoride membranes (Millipore, Billerica, MA, USA).

Immunoblotting was performed as follows. The membranes were subjected to bovine serum albumin treatment for 1 h at room temperature, and the membranes were incubated overnight at 4 °C with primary antibodies. The primary antibodies were cleaved caspase-3 (monoclonal anti-rabbit antibody; ab32042, Abcam, Cambridge, UK), Bcl-2 (monoclonal anti-rabbit antibody; ab32124, Abcam, Cambridge, UK), Bax (monoclonal anti-rabbit antibody; ab205822, Abcam, Cambridge, UK), beta-actin (monoclonal anti-mouse antibody; 66009, Proteintech, Rosemont, IL, USA), HMGB-1 (polyclonal anti-rabbit antibody; 6893, Cell Signaling Technology, Danvers, MA, USA), TLR4 (polyclonal anti-rabbit antibody; GTX21436, GeneTex, Irvine, CA, USA), NF-κB (p65) (polyclonal anti-rabbit antibody; ab16502, Abcam, Cambridge, UK), IκBα (monoclonal anti-mouse antibody; 4814, Cell Signaling Technology, Danvers, MA, USA), and pIκBα (mono-clonal anti-rabbit antibody; 2859, Cell Signaling Technology, Danvers, MA, USA). The secondary antibodies used were anti-mouse antibody (IgG antibody; 7076, Cell Signaling Technology, Danvers, MA, USA) and anti-rabbit antibody (IgG antibody; 7074, Cell Signaling Technology, Danvers, MA, USA). After using enhanced chemiluminescence solution (Advansta, San Francisco, CA, USA), bands were quantified using ImageJ software (version 1.53 q; National Institute of Health, Betheda, MD, USA).

### 2.5. ELISA for Estradiol

After treatment with 100 or 200 μM of H_2_O_2_ for 2 h, 1 × 10^5^ cells/well of hGL5 cells in 6-well plates were incubated in serum-free DMEM for 24 h. The cell supernatant was harvested, and the collected supernatant was analyzed for estradiol levels using an Estradiol Parameter Assay Kit (Bio-Techne, Minneapolis, MN, USA).

### 2.6. qRT-PCR for HMGB-1 and TLR4

According to the manufacturer’s instructions, total RNA was isolated from hGL5 cells using Trizol reagent (Invitrogen, Carlsbad, CA, USA). RNA concentration and purity were measured with spectrophotometer at A260 and A260/280, respectively. Moreover, according to the manufacturer’s instructions, RNA was reverse-transcribed into cDNA using Primescript™ RT reagent kit (Takara Bio, Kusatsu, Japan). The sequences of primers used were as follows: for GAPDH: forward, 5′-TCGACAGTCAGCCGCATCTTCTTT-3′ and reverse 5′-ACCAAATCCGTTGACTCCGACCTT-3′; for HMGB-1: forward, 5′-CAGGGCCAAACCGATAGGAAA-3′ and reverse, 5′-TCGTGCACCGAAAGTTTCAA-3′; for TLR4: forward, 5′-CAGAGTTTCCTGCAATGGATCA-3′ and reverse, 5′-GCTTATCTGAAGGTGTTGCACAT-3′. GAPDH was used as an internal control for evaluating the relative expressions of HMGB-1 and TLR4. qRT-PCR was performed on Veriti™ 96-Well Thermal Cycler (Thermo Fisher Scientific, Inc., Waltham, MA, USA) with the following thermocycling conditions: Initial denaturation at 95 °C for 30 s, followed by 32 cycles at 55 °C for 30 s and 72 °C for 30 s. A mixture of 10 µL SYBR™ Green PCR Master Mix (Thermo Fisher Scientific, Inc., Waltham, MA, USA) was used.

### 2.7. ELISA for IL-1β and IL-6

After treatment with 500 to 2000μM of H_2_O_2_ for 2 h, 3 × 10^5^ cells/well of hGL5 cells in 12-well plates were incubated in serum-free DMEM for 24 h. The collected supernatant was analyzed for IL-1β and IL-6 levels using Human Interleukin-1β (IL-1β) ELISA kit (ab214025, ABCAM, Cambridge, UK) and Human Interleukin-6 (IL-6) ELISA kit (ab178013, ABCAM, Cambridge, UK).

### 2.8. Statistical Analysis

Student *t*-tests with Bonferroni adjustment were performed to determine the differences in the cell count, mRNA, protein expression levels, estradiol secretion levels, and inflammatory molecule release levels. Linear regression analyses were used to investigate the linear association between cell proliferation, mRNA expression, estradiol secretion, inflammatory molecule release, and the concentration or duration of H_2_O_2_ treatment, allowing assessment of dose- and time-dependent effects. SPSS Statistics (version 28; IBM, New York, NY, USA) and R software (version 4.1.2; www.r-project.org (accessed on 10 April 2023); R Foundation for Statistical Computing, Vienna, Austria) were used for all statistical analyses. Statistical significance was set at *p*-value < 0.05.

## 3. Results

### 3.1. hGL5 Cell Proliferation After H_2_O_2_ Treatment

**Figure 1:**
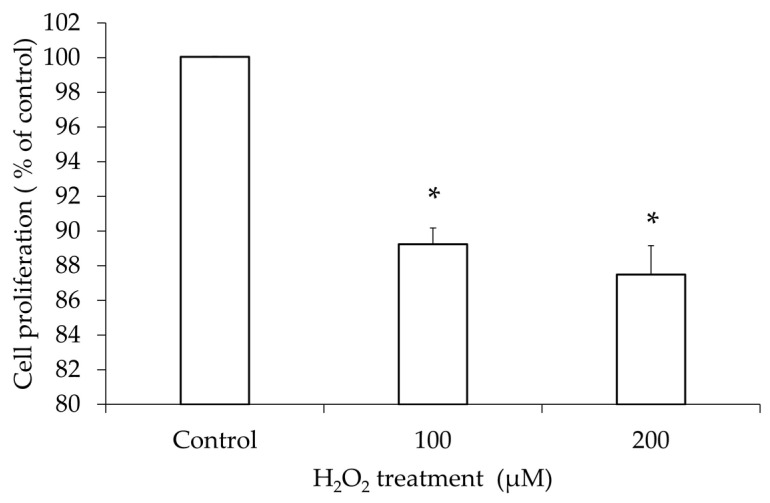
Proliferation of hGL5 cells after H_2_O_2_ treatment. Results of the cell counting kit-8 assay observed at OD 450 nm showed that the cell count decreased in an H_2_O_2_ concentration-dependent manner (R^2^ = 0.91, *p*–value for trend < 0.001 using linear regression model). These results are representative of three experiments. * *p*-value < 0.05 compared to the phosphate-buffered saline-treated control using two-tailed Student’s *t*-test.

To determine the effect of oxidative stress on the cell viability of granulosa cells, the change in viable cell count was examined by varying concentrations of H_2_O_2_ treatment to hGL5 cells using the CCK-8 assay. With H_2_O_2_ treatment of 100 μM and 200 μM, the cell count decreased compared to that of the control treated with PBS. Linear regression analysis showed significant an inverse correlation between the concentration of H_2_O_2_ and cell proliferation (Figure 1).

### 3.2. Apoptosis in hGL5 Cell After H_2_O_2_ Treatments

**Figure 2:**
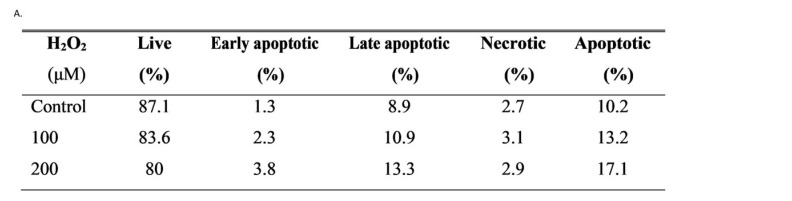

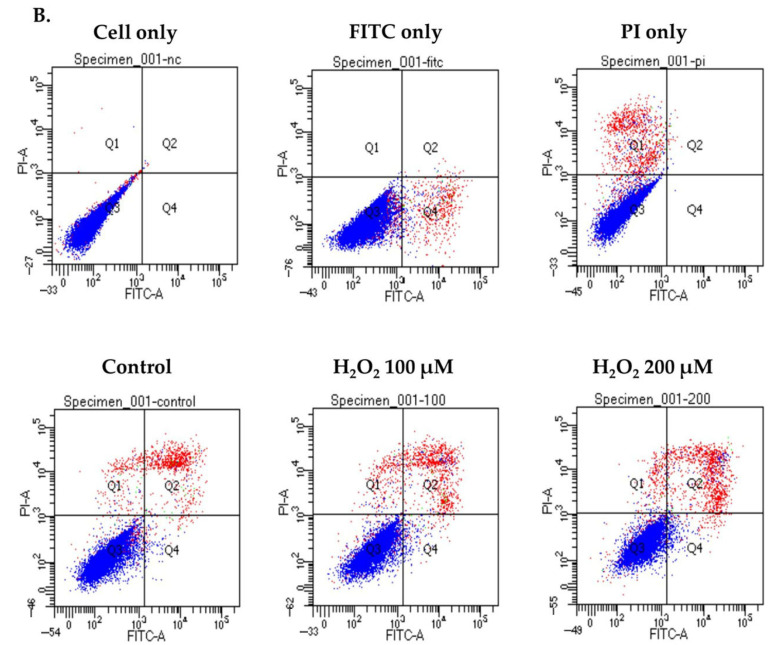
Apoptosis of hGL5 cells after H_2_O_2_ treatment. The proportion of early and late apoptotic hGL5 cells out of the entire cell count after 100 μM and 200 μM of H_2_O_2_ treatment increased compared to that of phosphate-buffered saline-treated control (**A**). The apoptotic hGL5 cells were identified by flow cytometry (**B**). (Live cells, Q3, AV−/PI−; cells in early stage of apoptosis, Q4, AV+/PI−; cells in late stage of apoptosis, Q2, AV+/PI+; cells undergoing necrosis, Q1, AV−/PI+). These results are representative of three experiments. AV, annexin V-FITC; PI, propidium iodide.

To gain insight into the effect of oxidative stress on the decreased proliferation of hGL5 cells, an apoptosis assay was performed after H_2_O_2_ treatment of 100 μM and 200 μM for 2 h. The proportion of live, early apoptotic, late apoptotic, and necrotic cells is expressed in Figure 2A. Early and late apoptotic cells increased as the concentration of H_2_O_2_ treatment increased. Because hGL5 cells were incubated with serum-free media for 24 h, the control also showed few apoptotic cells as a baseline. In flow cytometry analysis, the cell population shifted from live to early and late apoptotic after H_2_O_2_ treatment with 100 μM and 200 μM compared to the control (Figure 2B).

**Figure 3:**
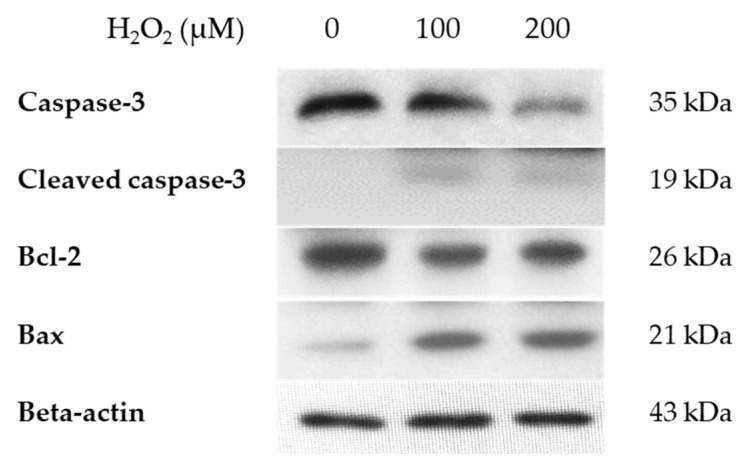
Western blot of the expression of apoptosis-related proteins in hGL5 cells after H_2_O_2_ treatment. The band of Bax and cleaved caspase-3 showed increased intensity, while that of caspase-3 and Bcl-2 showed decreased intensity as the concentration of H_2_O_2_ increased. These results are representative of three experiments.

A Western blot was performed to detect the expression of apoptosis-related proteins at the same time as cell death increased (Figure 3). Differing concentrations of H_2_O_2_-treated hGL5 cells were treated for 2 h and harvested after 24 h of incubation. After H_2_O_2_ treatment of various concentrations, the expression of caspase-3 decreased, but the expression of cleaved caspase-3 increased with low intensity after H_2_O_2_ treatment. The expression of pro-apoptotic protein Bax increased, whereas that of the anti-apoptotic protein Bcl-2 decreased.

### 3.3. Estradiol Secretion in the hGL5 Cell Supernatants After H_2_O_2_ Treatment

**Figure 4:**
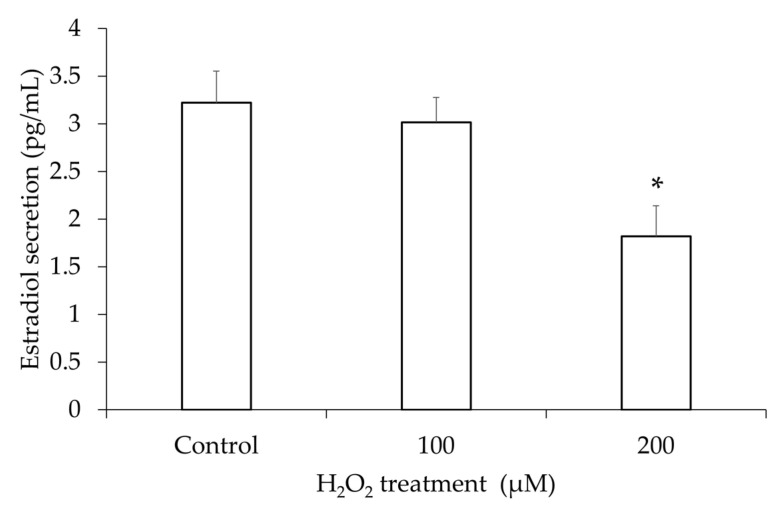
Estradiol in supernatant after H_2_O_2_ treatment. The secretion of estradiol by hGL5 cells exhibited a significant dose-dependent decrease in correlation with increasing H_2_O_2_ concentrations (R^2^ = 0.65, *p*-value for trend < 0.05 using linear regression model). These results are representative of three experiments. * *p*-value < 0.05 compared to the phosphate-buffered saline-treated control using two-tailed Student’s *t*-test.

Because one of the main functions of granulosa cells is steroidogenesis, increased cell death may decrease estradiol production and secretion. After inducing oxidative stress with H_2_O_2_, ELISA for estradiol was performed with the supernatant. The estradiol level after 200 µM of H_2_O_2_ treatment was significantly lower than that of the PBS-treated control (Figure 4). Although 100 µM of H_2_O_2_ treatment did not show a significant decrease, linear regression analysis showed a decreasing tendency of estradiol secretion in the supernatant after H_2_O_2_ in a dose-dependent manner.

### 3.4. mRNA and Protein Expression of HMGB-1 After H_2_O_2_ Treatment

**Figure 5:**
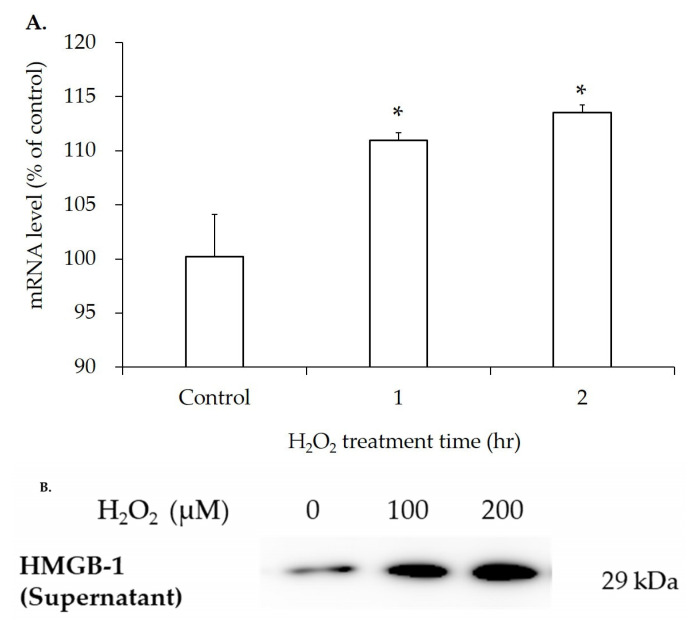
Relative mRNA level of HMGB-1 after H_2_O_2_ treatment: 200 µM of H_2_O_2_ was treated for different time periods and the mRNA level was analyzed. The mRNA level of HMGB-1 significantly increased after H_2_O_2_ treatment in time-dependent manner (R^2^ = 0.74, *p*-value for trend < 0.05 using linear regression model) (**A**). Western blot for HMGB-1 after H_2_O_2_ treatment was performed. The passive, extracellular release of HMGB-1 increased as the concentration of H_2_O_2_ increased (**B**). These results are representative of three experiments. HMGB-1, high-mobility group box-1. * *p*-value < 0.05 compared to the phosphate-buffered saline-treated control using two-tailed Student’s *t*-test.

To examine HMGB-1 changes in hGL5 cells after adding oxidative stress, qRT-PCR and Western blotting were performed. First, 200 µM H_2_O_2_ was treated for 1 and 2 h. The mRNA level of HMGB-1 in cell lyses increased after H_2_O_2_ treatment in a time-dependent manner (Figure 5A). Western blot for HMGB-1 was performed in the supernatant of hGL5 cells harvested after 2 h of H_2_O_2_ treatment and 24 h of incubation. Western blot showed increased extracellular HMGB-1 release in the supernatant according to the increased cell death induced by the increased dose of H_2_O_2_ treatment (Figure 5B).

### 3.5. Increased TLR4 mRNA Expression in hGL5 After H_2_O_2_ Treatment

**Figure 6:**
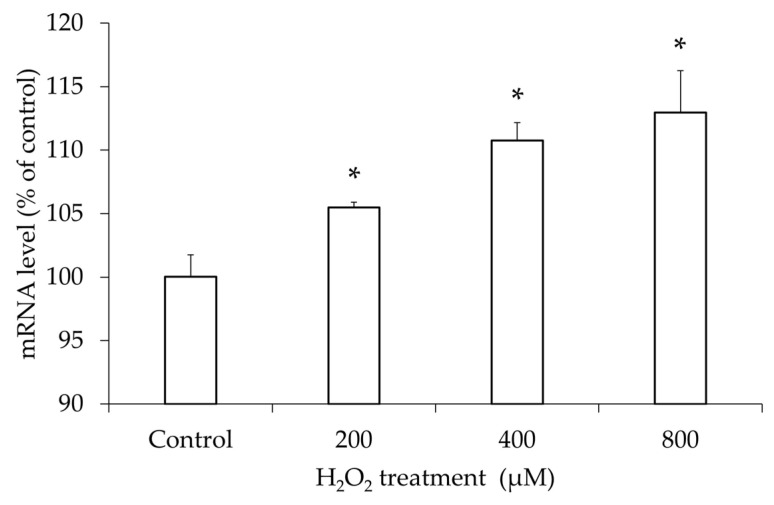
Relative mRNA level of TLR4 after H_2_O_2_ treatment. Relative mRNA level of TLR4 after treatment with different concentrations of H_2_O_2_ for 2 h was analyzed. The mRNA level significantly increased with all concentrations of H_2_O_2_ in dose-dependent manner (R^2^ = 0.61, *p*-value for trend < 0.05). These results are representative of three experiments. TLR4, toll-like receptor 4. * *p*-value < 0.05 compared to the phosphate-buffered saline-treated control using two-tailed Student’s *t*-test.

TLR4, a receptor of HMGB-1, was examined in hGL5 cells after oxidative stress. The mRNA level of TLR4 was analyzed with qRT-PCR after treatment with different concentrations of H_2_O_2_. Compared to the PBS-treated control, the mRNA level was significantly increased after H_2_O_2_ treatment in a dose-dependent manner (Figure 6).

### 3.6. Activation of NF-κB Pathway After H_2_O_2_ Treatment

**Figure 7:**
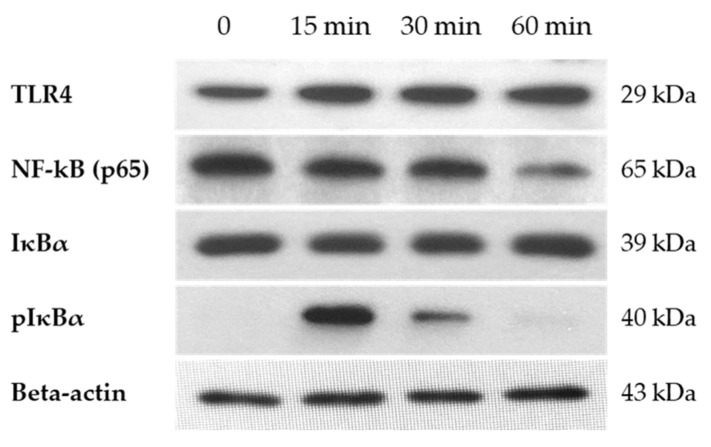
Western blot for TLR4 and NF-κB pathway-related proteins. Western blot was performed at 0, 15 min, 30 min, and 60 min after 200 µM H_2_O_2_ treatment. TLR4 showed increased protein synthesis compared to the control (at 0). NF-κB (p65) showed a gradual decrease in a time-dependent manner; pIκBα showed an increase at 15 min of treatment, gradually decreasing after. IκBα did not show any difference as time passed. These results are representative of three experiments. TLR4, toll-like receptor.

To examine the relationship between oxidative stress and NF-κB pathway activation in hGL5 cells, we treated H_2_O_2_ at a fixed dose of 200 µM, increasing the treatment time. We assessed the protein synthesis of TLR4 in hGL5 cell lyses immediately after media change as a baseline to 60 min after to catch the beginning of the signaling. In hGL5 cell lyses, phosphorylated IκBα (pIκBα) showed a peak increase 15 min after H_2_O_2_ treatment. Phosphorylation of IκBα liberates NF-κB proteins to translocate to the nucleus, activating the NF-κB pathway. Because the experiment was performed with whole lysates, only the gradual decrease in NF-κB (p65) as time passed due to degradation after its activation was observed (Figure 7).

### 3.7. Increased Release of Inflammatory Cytokines

**Figure 8:**
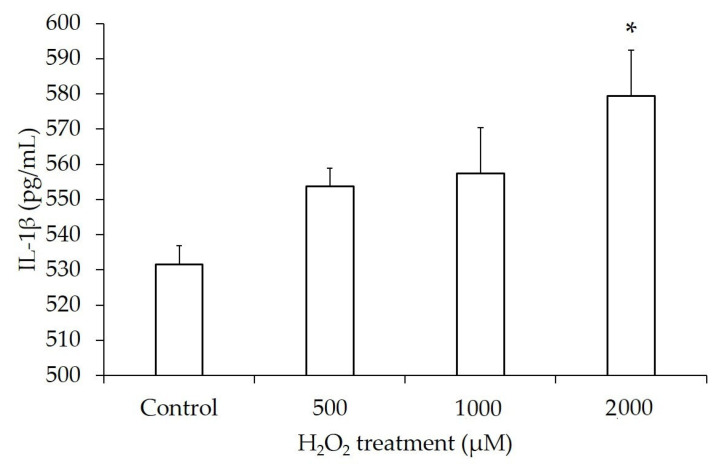
IL-1β secretion after H_2_O_2_ treatment. The level of IL-1β was significantly increased at 2000 µM of H_2_O_2_ compared to the control. The linear regression analysis showed an increasing tendency of IL-1β in a dose-dependent manner (R^2^ = 0.56, *p*-value for trend < 0.05). These results are representative of three experiments. IL-1β, Interleukin-1β. * *p*-value < 0.05 compared to the phosphate-buffered saline-treated control.

We measured proinflammatory cytokines in the supernatant of H_2_O_2_-treated hGL5 cells to determine the final outcome of the chain reaction resulting from oxidative stress-induced NF-κB pathway activation. It was difficult to detect the cytokine secretion under the same conditions of our experiment as before. Therefore, we increased the hGL5 cell concentration and the dose of H_2_O_2_. IL-1β was significantly increased after treatment with 2000 µM H_2_O_2_ for 2 h. Even though the significance was shown at 2000 µM only, there was a significant increasing tendency of IL-1β in a dose-dependent manner analyzed by linear correlation analysis (Figure 8).

**Figure 9:**
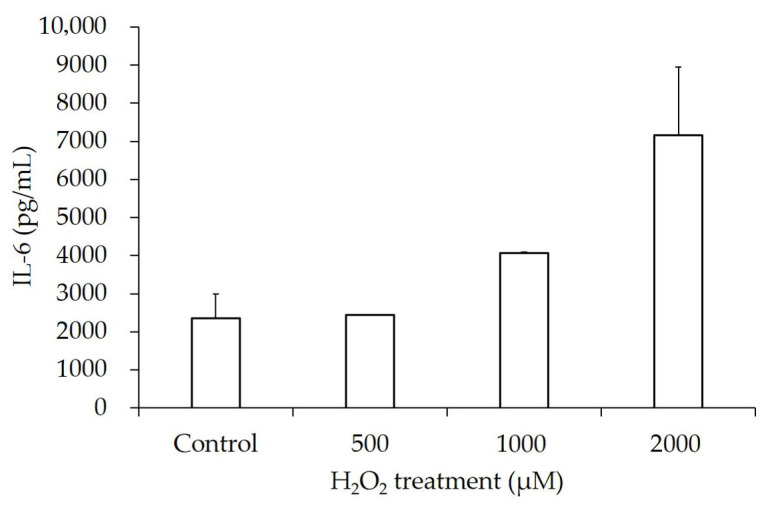
IL-6 secretion after H_2_O_2_ treatment. The secretion of IL-6 after H_2_O_2_ treatment was at a peak with the highest H_2_O_2_ concentration of 2000 µM, but the difference compared to the phosphate-buffered saline-treated control with was not significant (*p*-value = 0.21). However, the IL-6 release increased in a dose-dependent manner (R^2^ = 0.64, *p*-value for trend < 0.05). IL-6, interleukin-6.

IL-6’s average concentration after H_2_O_2_ treatment was not significantly increased compared to that of the PBS-treated control. However, it showed a dose-dependent trend as the dose of H_2_O_2_ treatment increased to 2000 µM (Figure 9).

Secretion levels of IL-1β and IL-6 in the hGL5 cell line increased in a dose-dependent manner with increasing H_2_O_2_ concentrations, and these results are summarized in Table 1.

To summarize, due to EMS, increased oxidative stress affects the viability and increases the apoptosis of granulosa cells. HMGB-1 release increases, contributing to a positive feedback loop amplifying oxidative stress. The oxidative stress also leads to increased expression of TLR4 sequentially and decreased steroidogenesis of granulosa cells. The interaction of HMGB-1 and its receptor, TLR4, activates the NF-κB pathway, and the release of inflammatory molecules IL-1β and IL-6.

## 4. Discussion

In the context of in vitro fertilization (IVF), EMS is associated with reduced oocyte yield, lower implantation rates, and decreased pregnancy success. By affecting oocyte yield and quality, EMS exerts a detrimental impact on the follicular environment, thereby impairing fertility. HMGB-1, a well-known danger signal, has been implicated in inflammation, cell proliferation, immunity, metabolism, cancer, and oxidative stress [17,18,19,20]. Studies on the pathophysiology of EMS have indicated that HMGB-1 participates in activating the NF-κB pathway and regulating inflammatory responses and autophagy in HESCs [16,21]. Similarly, Gonzalez-Ramos et al. demonstrated the activation of the NF-κB pathway by proinflammatory cytokines contributes to the progression of EMS [22]. Despite these insights, the role of HMGB-1 in infertility is unknown. To the best of our knowledge, the present study is the first to report the role of HMGB-1 associated with granulosa cells.

hGL5 cell proliferation was reduced due to apoptosis, which also resulted in decreased estradiol secretion. In assisted reproductive technology (ART), the quality of oocytes and embryos is one of the most critical parameters determining pregnancy outcomes. Since oocytes are surrounded by granulosa cells, these cells play an essential role in follicle development, fertility, and oocyte viability. Not only the quantity but also the function of granulosa cells, particularly in terms of steroidogenesis, is important [23,24]. Decreases in estradiol levels in EMS patients are associated with reduced IVF outcomes, including pregnancy and live birth rates [25,26]. Therefore, diminished steroidogenesis due to granulosa cell apoptosis may negatively affect fertilization.

HMGB-1 release increased after oxidative stress in the hGL5 cell line as HMGB-1 release increased in HESCs by oxidative stress-induced cell death [16]. Also, increased TLR4 expression was observed. The binding of HMGB-1 to TLR4 may lead to the worsening of oxidative stress in EMS. It is rather well known that HMGB-1-TLR-4 interaction stimulates inflammatory reaction in various cells [27,28].

Clinical studies have demonstrated that women with EMS exhibit altered serum and local cytokine profiles characterized by increased levels of pro-inflammatory cytokines such as IL-1β, IL-6, IL-8, and TNF-α, as well as elevated anti-inflammatory cytokines including IL-4 and IL-10, reflecting a dysregulated inflammatory environment [29,30]. The expression of IL-1β and IL-6 after H_2_O_2_ treatment was also increased in the hGL5 cell line in this study. Huang et al. demonstrated that increased HMGB-1 levels are associated with the enhanced release of inflammatory cytokines, including IL-1β and IL-6 [21]. Activation of the NF-κB pathway following oxidative stress may represent the link between HMGB-1 and inflammatory cytokine production, highlighting the pivotal role of the NF-κB pathway in EMS [31,32]. In Western blot analysis of NF-κB pathway-related proteins, IκBα levels remained unchanged over time, whereas its phosphorylated form peaked at 15 min post-treatment and gradually decreased thereafter. This is likely due to cytoplasmic localization of IκBα and subsequent NF-κB nuclear translocation resulting from ubiquitination of IκB [33]. Along with decreased steroidogenesis, increased release of IL-1β and IL-6 may partly explain the low quality of oocytes in EMS. Further studies are warranted to explore this possibility.

Our study has shown that oxidative stress due to EMS affects the follicular environment via the apoptosis of granulosa cells, decrease in estradiol secretion, the cascade involving HMGB-1, TLR4, the NF-κB pathway, and the release of inflammatory molecules IL-1β and IL-6.

The limitation of this study is that the results were only from an in vitro cell-level perspective. Therefore, it may not fully capture the complex environment of ovarian follicles in vivo. Confirming the involvement of the HMGB-1-TLR4-NF-κB axis through in vivo experiments with actual follicles is necessary. Also, the variability and heterogeneity of EMS in patients were not reflected in our cell-based model. Clinical differences could influence the observed molecular pathways differently. We focused on major inflammatory cytokines such as IL-1β and IL-6 in relatively higher concentrations of H_2_O_2_. However, many other factors may contribute to EMS-related infertility. Future research with human clinical samples is essential to validate the clinical relevance of the HMGB-1/TLR4/NF-κB axis in EMS.

## 5. Conclusions

This study showed increased HMGB-1 release, decreased steroidogenesis, and increased inflammation in granulosa cells after oxidative stress to explain the follicular environmental changes in patients with EMS and infertility. These findings can be suggested as a new research topic and therapeutic target for such patients.

## Figures and Tables

**Table 1:** IL-1β and IL-6 secretion after H_2_O_2_ treatment. The level of IL-1β was significantly increased at 2000 µM of H_2_O_2_ compared to the control.

H_2_O_2_ Concentration	IL-1β (pg/mL)	*p*-Value	IL-6 (pg/mL)	*p*-Value
Control	531.62 ± 10.40		2355.05 ± 1270.04	
500 µM	553.68 ± 10.40	0.08	2445.90 ± 25.02	0.46
1000 µM	557.35 ± 26.00	0.16	4075.57 ± 51.92	0.10
2000 µM	579.41 ± 26.00	<0.05	7163.67 ± 3570.18	0.11

## Data Availability

The datasets used and/or analyzed during the current study are available from the corresponding author on reasonable request.
